# Properties of Lightweight Aggregate Concrete Reinforced with Carbon and/or Polypropylene Fibers

**DOI:** 10.3390/ma13030640

**Published:** 2020-01-31

**Authors:** Hui Wei, Tao Wu, Xue Yang

**Affiliations:** School of Civil Engineering, Chang’an University, Xi’an 710061, China; weihuichd@163.com (H.W.); ms_yangxue@163.com (X.Y.)

**Keywords:** lightweight aggregate concrete, mechanical properties, stress–strain behavior, toughness, carbon fiber, polypropylene fiber

## Abstract

The impact of carbon and polypropylene fibers in both single and hybrid forms on the properties of lightweight aggregate concrete (LWAC), including the slump, density, segregation resistance, compressive strength, splitting tensile strength, flexural strength, and compressive stress–strain behavior, were experimentally investigated. The toughness ratio and ductility index were introduced for quantitatively evaluating the energy-absorbing capacity and post-peak ductility. A positive synergistic effect of hybrid carbon and polypropylene fibers was obtained in terms of higher tensile strength, toughness, and ductility. The toughness ratio and ductility index of hybrid fiber-reinforced LWAC were increased by 26%–37% and 12%–27% compared with plain LWAC, respectively. The fiber in both single and hybrid forms had a smaller effect on the linearity ascending branch of the stress–strain curves, whereas the post-peak patterns in terms of the toughness and ductility for the hybrid fiber-reinforced LWAC were significantly improved when the fiber in hybrid form.

## 1. Introduction

The utilization of lightweight aggregate concrete (LWAC) in the construction industry has increased rapidly owing to its unique economic and technical benefits, including a lower density, higher specific strength, and better durability [1,2,3]. The reduction in the dead load achieved through the low density of LWAC not only decreases the cross-section of the structural components, but also reduces the risk of earthquake damage to a structure [4]. Nevertheless, the lower particle strength of an aggregate allows cracks to propagate across the lightweight aggregate (LWA) rather than around it. Hence, LWAC exhibits greater brittleness than normal weight concrete (NWC) at a comparable strength, which limits the further promotion of LWAC in structural applications [5].

Effective enhancement of the overall performance of concrete can be achieved through the incorporation of fibers. Tanyildizi [6] used carbon fibers to reinforce lightweight concrete and claimed that both the compressive and flexural strengths of concrete were enhanced by carbon fiber. Li et al. [7] reported that polypropylene fiber effectively reinforced the LWAC and improved its tensile strength, flexural toughness, and impact resistance; moreover, an optimal polypropylene fiber content of 9 kg/m^3^ was recommended. Although a single fiber improves the properties of concrete at only a single scale, it was reported that concrete reinforced with two or more different types of fiber can benefit from each individual type of fiber and generate a positive synergistic response [8]. For hybrid fiber-reinforced concrete, dispersed fibers can effectively restrain the formation and propagation of cracks through a bridging effect at both the macro and micro levels, leading to an increase in the tensile behavior and toughness [9]. Guler [10] reported that a combination of different types of polyamide synthetic fiber is more effective in improving the strength and toughness of LWAC than single fiber only. Smarzewski [11] pointed out that basalt and polypropylene fibers can have an effect at different deformation stages of concrete. The strengthening and toughening brought by hybrid basalt-polypropylene fibers have been attributed to the prevention of crack formation and propagation. Aslani et al. [12] used steel and polypropylene fibers to reinforce concrete, and found that an appropriate hybrid ratio can increase the strength and toughness of concrete. Steel fiber being used individually or combined with synthetic fiber is the most commonly used type of fiber for improving the mechanical properties of concrete [13]. Considering its high specific gravity, the addition of steel fiber has an adverse effect on the lightweight characteristic of LWAC, increasing the dead load of a composite. Gao et al. [14] showed that the addition of steel fiber in volume fractions of 1%, 1.5%, and 2% increases the density of LWAC by 4.6%, 5%, and 7.5%, respectively.

Synthetic fibers such as carbon and polypropylene fibers have attracted significant attention owing to their high performance and low specific gravity [15,16]. However, the mechanical properties of LWAC reinforced with only carbon and carbon/polypropylene fibers has yet to be elucidated. Carbon fiber is a type of high-modulus fiber that possesses high strength and excellent resistance to corrosion and high temperatures [17,18]. In particular, short carbon fiber is easy to distribute uniformly in concrete and in turn results in isotropic mechanical properties [19]. Polypropylene fiber has high ductility, chemical stability, and a low-modulus, and has been recognized as the most typically used synthetic fiber [20,21]. A large number of polypropylene fibers crossing the cracks in a concrete mixture can prevent and control the propagation of cracks and limit the crack width [22], which benefits the tensile behavior and toughness of the concrete. Hence, it is believed that the hybridization of high-modulus carbon fiber with high ductility polypropylene fiber can hope to reinforce the performance of concrete at multiple scales, thereby enhancing the tensile strength, toughness, and ductility of the LWAC.

In this study, eight combinations of carbon and polypropylene fiber content by volume (carbon/polypropylene = 0.2/0, 0.4/0, 0/0.2, 0/0.4, 0.2/0.2, 0.2/0.4, 0.4/0.2, 0.4/0.2%) were used to manufacture LWAC. The properties of LWAC reinforced with carbon and/or polypropylene fibers, including the density, slump, segregation resistance, cube compressive strength with elapsed age, splitting tensile strength, and flexural strength, were experimentally investigated. Moreover, the stress–strain relationship based on the axial compression test was also investigated. Both the toughness ratio and ductility index were introduced to quantitatively analyze the effect of the fiber on the post-peak behavior of the curve, as well as the toughness and deformability of the concrete mixture.

## 2. Experimental Investigation

### 2.1. Mix Proportions and Materials

To investigate the effect of fiber reinforcement on the mechanical properties of lightweight expanded shale aggregate concrete, a control mixture with no fiber addition and four mixtures with a single fiber reinforcement were formulated using carbon or polypropylene fibers. Next, four additional mixtures of LWAC containing carbon-polypropylene hybrid fibers were adopted to examine the synergistic effect of the fiber addition. Table 1 shows the fiber dosages of all nine mixtures. Both carbon and polypropylene fibers at volume fractions of 0.2% and 0.4% were used. Each of these mixtures had the same amount of mineral admixtures, aggregate, and water. For the mineral admixtures, the mass percentages of cement, fly ash, and silica fume were 80%, 12%, and 8%, respectively. The effective water-to-binder ratio (w/b) was fixed at 0.30 by mass to achieve dense packing. The mixture composition of plain LWAC is shown in Table 2.

**Figure 1:**
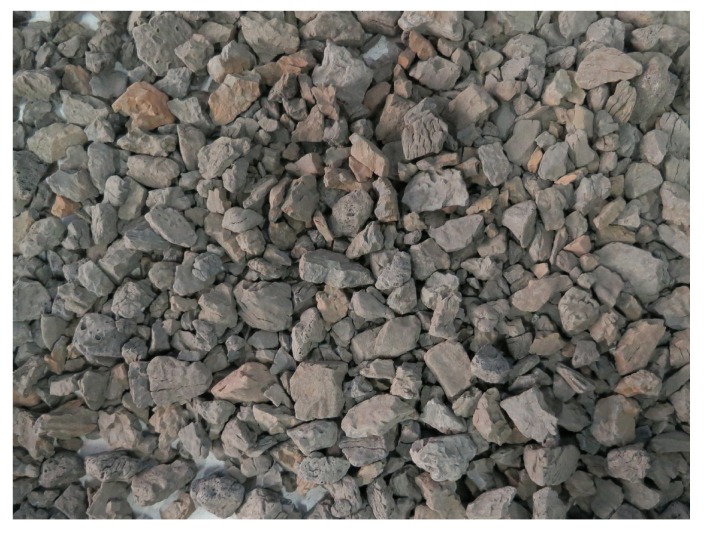
Angular expanded shale ceramist.

**Figure 2:**
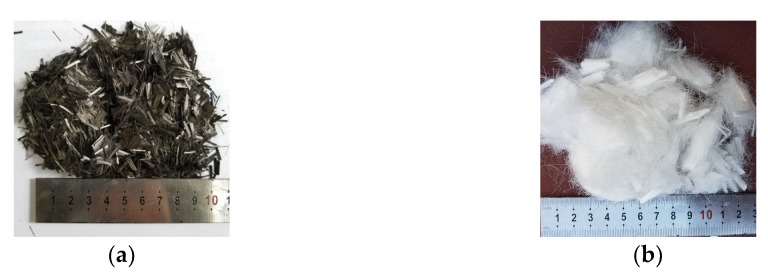
Fiber features: (**a**) carbon fiber, (**b**) polypropylene fiber.

In this study, for fabricating the fiber-reinforced LWAC specimens, an angular expanded shale ceramist (Figure 1) from Hubei, China was used as the coarse aggregate in all mixtures. Table 3 lists the physical properties and size distribution of the LWA. This LWA was characterized by a vitreous shell outside and a porous structure inside. Pores inside the aggregate were generally independent of each other, which suggests a significant reduction in water absorption (24 h water absorption of 2.6%) as well as a high crushing strength of the aggregate [23]. Two types of fiber (carbon and polypropylene) were selected, as shown in Figure 2. The diameters of the carbon and polypropylene fibers were approximately 7 and 80 μm, respectively. The mechanical properties and geometric information of these two fibers, as provided by the manufacturers, are listed in Table 4.

The cement used was ordinary Portland cement (P. O 42.5) in compliance with the relevant Chinese Standard GB 175-2007 [24]. Two types of mineral additive, namely class F fly ash and a silica fume, were adopted owing to their environmental friendliness and pozzolanic properties [25]. Table 5 presents the chemical composition of the cement, silica fume and fly ash. The fine aggregate was natural river sand with a maximum size and specific gravity of 4 mm and 2.62 g/cm^3^, respectively. A polycarboxylic-based high-range superplasticizer was employed as an admixture to improve the fiber distribution and fluidity in the fresh mixture [26].

### 2.2. Specimen Preparation

The LWA was moistened in advance with additional water and allowed to stand for 24 h to compensate for its water absorption. To guarantee the dispersion of the fiber in the mixture, the carbon fiber should be blended with a partial mixture of water, using polypropylene fiber added directly to the mixture. The mixing procedures for the fiber-reinforced LWAC were as follows. First, cement, fly ash, silica fume, and fine aggregate were added into a double-axis mixer and dry-mixed for 2 min. Polypropylene fiber was added by hand and mixed for an additional 2 min. Then, 50% of the premixed water with mixed superplasticizer was gradually poured into the dry solid materials and mixed for 3 min followed by the addition of pre-wetted LWA and a further mixing for another 5 min. Then, carbon fiber and the remaining water were then mixed together and slowly added into the mixer. Another 3–5 min of mixing was needed to complete the mixing procedure, taking care to obtain a homogenous mixture. At this stage, the slump value of the fresh LWAC mixture was measured immediately, consistent with the Chinese Standard GB 50164-2011 [27]. Fresh concrete was then cast into the mold and vibrated using a vibrating table. The specimens were covered with a plastic sheet and placed at room temperature to prevent evaporation. De-molding was conducted after 24 h and the specimens were then cured in water at a temperature of 23 ± 3 °C until reaching the testing age or for an additional 28 days.

### 2.3. Test Setup and Procedure

#### 2.3.1. Segregation Resistance

**Figure 3:**
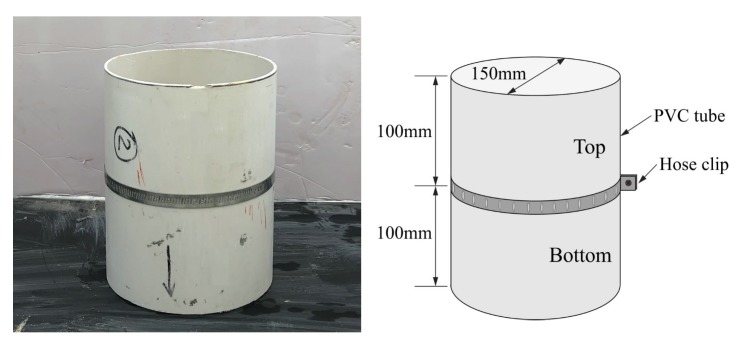
Segregation resistance test apparatus.

The segregation resistance was tested to characterize the stability of the LWA in the LWAC in accordance with the method suggested by Daneti and Wee [28]. As shown in Figure 3, the test apparatus consisted of two 150 mm × 100 mm equal PVC tubes and fastened in place with a leak-free hose clip while vibrating. First, the fresh LWAC mixture was poured into the segregation mold and subjected to a vibrating table for 2 min. Two portions of the cylinder mold were then separated horizontally after standing for approximately 30 min. The coarse aggregate in each portion was washed out through a 6 mm sieve and then oven-dried at a temperature of 105 °C for a sufficient amount of time to reach a constant mass. The segregation resistance of the LWAC can be represented mathematically based on the segregation coefficient (*SC*) and mass deviation index (*MI*).

The segregation coefficient can be estimated using a sum of squares approach through the following equation [29]:

(1) $$SC=\sqrt{\frac{h\sum_{i=1}^{n}{\left(\chi_{i}-\bar{\chi }\right)}^{2}}{H}}\times 100\%$$

where *SC* is the segregation coefficient (%), *h* is the height of each layer (*h* = 100 mm in this study), *H* is the total height of the test mold (*H* = 200 mm), *n* is the number of layers in the mold (*n* = 2), $\chi_{i}$ is the ratio of the mass of the coarse aggregate in each layer to the total mass of the coarse aggregate in the entire mold (%), and $\bar{\chi }$ is the weighted mean of the coarse aggregate (*χ* = 50%).

The mass deviation index can be expressed as follows:

(2) $$MI=\frac{1}{n}\sum_{i=1}^{n}\left|\frac{m_{i}-\bar{m}}{\bar{m}}\right|\times 100\%$$

where *MI* is the mass deviation index (%), *m_i_* is the mass of the oven-dried coarse aggregate in each layer (g), and $\bar{m}$ is the average coarse aggregate mass of the two layers (g).

#### 2.3.2. Mechanical Properties

The mechanical property tests and corresponding specimen details for each mixture are summarized in Table 6. The oven-dry density was measured at 28 days in accordance with the Chinese Standard JGJ 51-2002 [30]. The mechanical property tests were conducted according to the Chinese Standards GB/T 50081-2002 [31] and CSCE 13: 2009 [32].

**Figure 4:**
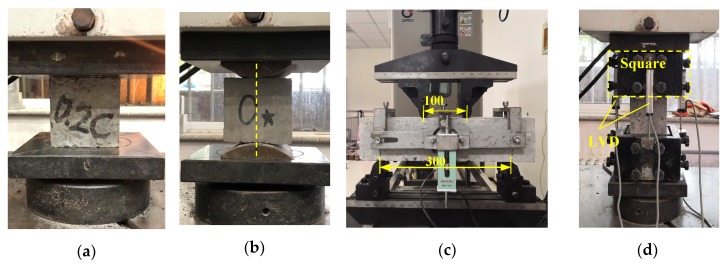
Mechanical property test setup for: (**a**) cube compressive strength, (**b**) splitting tensile strength, (**c**) flexural strength, (**d**) stress–strain curve.

For each concrete mixture, eighteen cubed specimens of 100 mm^3^ in size were used to determine the cubic compressive strength at 3, 7, 28, 60, 120, and 240 days, and three 100 mm^3^ specimens and three prismatic beams (100 × 100 × 400 mm^3^) were tested to determine the splitting tensile strength and flexural strength at 28 days, respectively. As shown in Figure 4, the compressive strength and splitting tensile strength tests were carried out on an electric-hydraulic servo universal testing machine (Changchun, China) with a 1000 kN and 300 kN capacity at a load rate of 6 and 1 kN/s, respectively. The flexural strength was conducted under four-point flexural tests with a 100 kN capacity electromechanical universal testing machine (CMT5105) at a loading rate of 0.01 kN/s

To obtain a complete and stable stress–strain curve, rigid square rings were installed at the end of the specimen to avoid local concrete crushing. The compression load was applied to the concrete prism through a displacement control at a slow rate of 0.01 mm/min. Moreover, the axial deformation was the average value of four linear variable displacement transducers (LVDTs) equipped on the sides of the square rings. A DH-3820 quasi-static data acquisition system running the TestExpert program was employed to record the LVDT value and corresponding load at a rate of once per second. To avoid an eccentricity of the loading, prior to the test, the specimen was pre-compressed to appropriately 30% of the compressive strength and unloaded to 0.5 MPa more than three times to eliminate the slackness of the system [33].

## 3. Test Results and Discussion

### 3.1. Slump

Table 7 shows the slump values of LWAC containing carbon and/or polypropylene fibers. The superplasticizer and water content were kept constant to evaluate the effect of different fiber combinations on the workability of fresh concrete. As shown in Table 7, a maximum slump of 255 mm was obtained using a plain mixture, whereas the C0.4PP0.2 mixture exhibited a minimum slump of 65 mm. This suggests that LWAC with a slump of 50–75 mm is comparable to that of NWC with a slump of 100–125 mm, which can obtain sufficient workability [34]. Accordingly, the workability of C0.4PP0.2 is comparable to that of NWC with a minimum slump of approximately 125 mm. Therefore, all tested concrete mixtures, with slump values within the range of 65–255 mm, were found to exhibit acceptable workability.

As can be observed, the incorporation of single carbon fibers, polypropylene fibers, and a hybrid of both reduced the slump by approximately 16%–60%, 2%–9%, and 16%–74%, respectively, compared with that of the plain mixture. As expected, the addition of fiber resulted in a negative effect on the workability of the fresh concrete, which might be associated with the confinement and obstructive effect of the fiber [35]. The highly specific surface area of carbon fiber and its hydrophilic surface may be another important reason. However, an exception to this occurred, in which the addition of polypropylene fiber did not significantly alter the slump value of fresh concrete, which can be observed in the results of the comparison between both PP0.2 and PP0.4 as well as C0.4PP0.2 and C0.4PP0.4. Similar results can be gained from the test results of Libre et al. [36]. The reason for this is attributed to the hydrophobic surface of the polypropylene fiber, which helps prevent a balling effect by the fiber. The water demand of polypropylene fiber in the mixed concrete is zero; moreover, with the addition of a superplasticizer, an adverse effect of polypropylene fiber on the workability can be overcome.

### 3.2. Density

**Figure 5:**
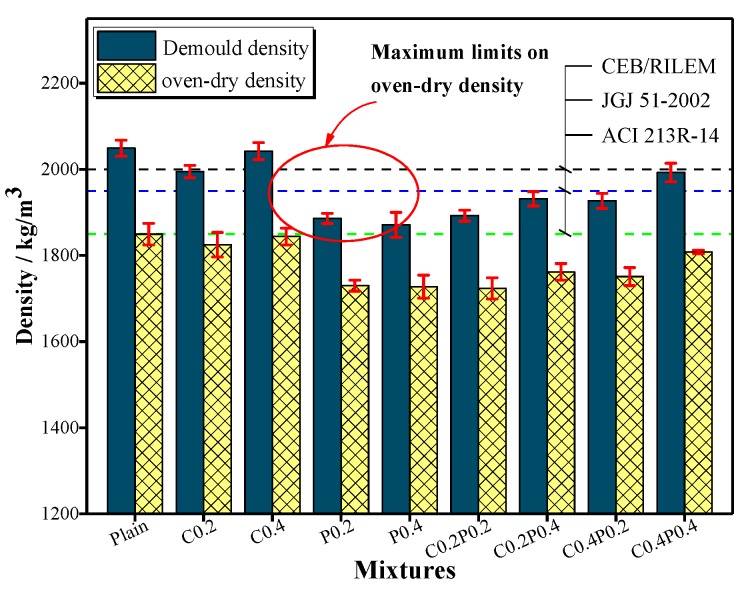
Density for LWAC reinforced with carbon and/or polypropylene fibers.

In some cases, the density of the concrete is more important than the strength, particularly for the lightweight concrete. A decreased density for a comparable level of strength enables a decreased dead load for the structural design. Two types of density, namely demolded density and 28-day oven-dry density, were measured and are presented in Table 7 and Figure 5. As can be observed, the demolded density and oven-dry density are within the range of 2049–1871 kg/m^3^ and 1849–1724 kg/m^3^, respectively. The demolded density of the concrete was approximately 150 to 200 kg/m^3^ higher than its oven-dry density. The demolded density varied with the absorbed moisture content as well as the degree of hydration, and thus, it is suggested that the oven-dry density was more representative than the demolded density. Figure 5 shows the upper limitation of the oven-dry density of LWAC based on several standards. The 28-day compressive strength of the concrete varies between 41 and 67 MPa for an oven-dry density of 1724–1849 kg/m^3^, which fulfills the requirements for structural LWAC with respect to the density and strength [37,38].

As shown in Figure 5, the incorporation of fibers into the LWAC reduced the density compared with the plain LWAC. The specimen consisting of 0.2% carbon fiber and 0.2% polypropylene fiber had the lowest density. Some researchers have attributed this to the fact that microfibers have lower specific gravity than cement paste [39,40,41]. However, as shown in Figure 5, the oven-dry density of LWAC containing a single carbon fiber showed a slight change in sensitivity compared with the plain LWAC. The reduction in the density is hardly illustrated the specific gravity of the fiber without a combination of extra factors. Victor [41] demonstrated that the fiber density is not critical in terms of the physical properties of the concrete when considering the use of a low fiber volume fraction. The main reason for the reduction in density may be the presence of entrapped air on the surface of the polypropylene fiber, and as a result, the formation of increased voids reduces the density of the hardened concrete. Referring to LWAC containing a single carbon fiber, the hydrophilic surface of the carbon fiber will promote a hydration reaction around the fiber, avoiding the entrapment of air on the fiber surface.

### 3.3. Stability and Segregation of LWA

According to ACI 116 R [42], segregation is defined as the non-uniformity of a concrete mass in the vertical direction, which results from the different concentrations of coarse aggregates and the cement paste of the concrete. In LWAC, a floating of coarse LWAs can be observed owing to their lower density. In this study, the bulk density of LWA was approximately 54% lighter than that of the plain counterpart.

Table 8 shows the distribution of coarse LWAs in the fresh concrete mixtures, from which the values of *SC* and *MI* are determined according to Equations (1) and (2). In general, the concentration of LWA at the top layer of the columns was larger than that of the bottom layer for all nine tested mixtures, proving the existence of an upward movement of LWA. The values of *SC* and *MI* of LWAC were within the range of 0.35%–1.38% and 0.69%–2.76%, respectively, which occurred within a narrow scope. As Chia and Zhang [43] claimed, concrete of *MI* ≥ 15% was considered to exhibit significant segregation between LWAs and the cement paste. All nine LWAC mixtures have a satisfactory resistance because they exhibit a mass deviation index, *MI*, of much smaller than the suggested limitation. The reason for the good segregation resistance could be related to the proportion of the components of mixed concrete. As Section 2.1 shows, the content in the cementitious materials was fixed at 550 kg/m^3^, occupying approximately 18% of the concrete mixtures by volume. In addition, the effective w/b employed was 0.30 for all mixtures. Accordingly, a satisfactory viscosity of the cement paste can be obtained, which presents the mixture from segregating, allowing stability of the LWA in the cement paste to be achieved.

The test results also indicate that both *SC* and *MI* are decreased when the fiber volume fraction is increased. It can be observed that the segregation potential of plain LWAC is higher than that of the LWAC containing fiber, and the segregation resistance of LWAC with hybrid fibers was found to be better than that with a single fiber. Accordingly, the segregation tendency of coarse LWAs can be suppressed through the addition of the fiber. Such behavior is attributed to the cohesive forces between the fiber and the cement paste resulting from a higher specific surface area of the fiber [44]. Moreover, the hybrid fibers can form a three-dimensional network structure in concrete, which effectively restrains the movability of the aggregate [45].

**Figure 6:**
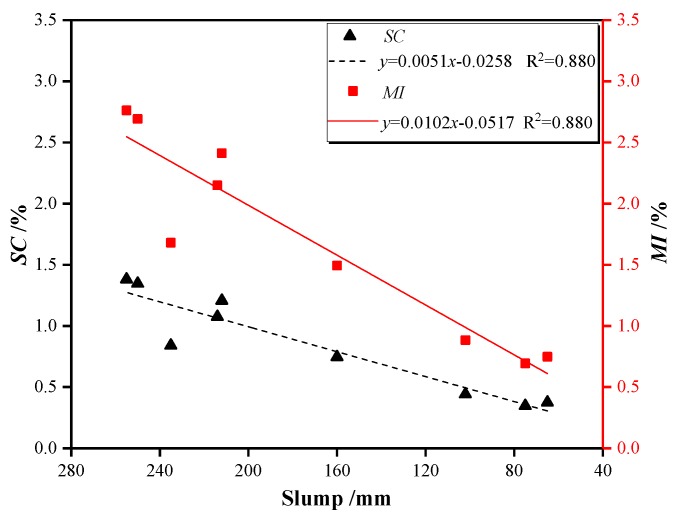
Relationship between the slump value and segregation parameters (*SC* and *MI*).

Comparing the results of the slump test with the segregation test, the segregation parameters almost keep in line with the changing trend of the slump values. The relationship between slump value and segregation parameters are graphically represented in Figure 6, which can be described by the first-order polynomials. Both *SC* and *MI* tend to decrease with the decrease of the slump value. The correlation coefficients of the fitting line are small enough to indicate a reasonable correlation between the slump and segregation parameters. This conclusion is consistent with the observation from Daneti and Wee [28], who reported the presence of a reasonable relationship between segregation resistance and workability.

### 3.4. Specific and Cube Compressive Strengths

**Figure 7:**
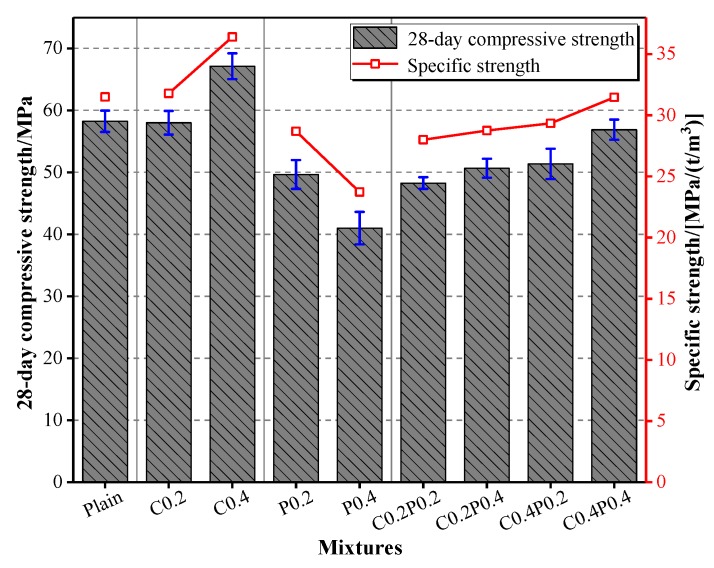
The 28-day compressive strength and corresponding specific strength of LWAC.

The compressive strength is related to the density of the specimen, and the specific strength (i.e., the ratio of compressive strength to the oven-dry density) is considered a good measure to characterize the high-strength and lightweight nature of LWAC. Figure 7 shows the variation of the 28-day cube compressive strength and the corresponding specific strength according to the fiber combinations. As can be observed, the variation tendency of the specific strength of LWACs was consistent with that of the compressive strength when varying the content of the fiber, through which the close relationship between the strength and density can be confirmed. The 28-day compressive strength of LWAC was within the range of 40.99 to 65.45 MPa for a specific strength of 23.72 to 35.49 MPa/(t/m^3^) when the LWAC was reinforced with 0.2% and 0.4% of carbon and/or polypropylene fiber. An increasing trend in both the compressive and specific strengths was shown with the incorporation of fiber, except for the concrete containing solely polypropylene fiber. The concrete reinforced with 0.4% polypropylene fiber exhibited the minimum compressive and specific strengths. Despite a reduction in the specific strength for the addition of polypropylene fiber, LWAC showed a higher specific strength (minimum value of 23.72 MPa/(t/m^3^)) than that of the NWC (18.63 MPa/(t/m^3^)) [46].

For concrete reinforced with a single fiber, the polypropylene fiber gave rise to a more adverse effect on the compressive strength than carbon fiber at the same volume fraction. This can be correlated with the higher elastic modulus and tensile strength of the carbon fiber, which is of greater efficiency in restraining the transverse deformation. The incorporation of fiber reduced the compressive strength of LWAC owing to that the microfibers decrease the consolidation of the concrete, and the compressive strength was therefore decreased [15].

**Figure 8:**
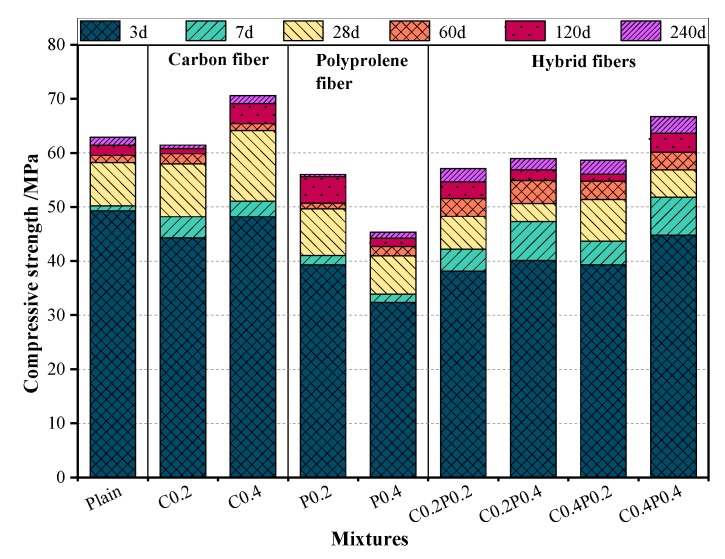
Development of cube compressive strength of LWAC.

The ranges of compressive strength after 3, 7, 28, 60, 120, and 240 days were found to be 32.3–49.3, 33.9–52.1, 41–65.5, 42.7–66.4, 44.3–72.1, and 45.3–73.6 MPa, respectively, as shown in Table 9. The rate of compressive strength development depending on the fiber combination and curing ages is shown in Figure 8. Apparently, the growth rate in the compressive strength for all nine LWAC mixtures exhibited a similar tendency. All mixtures were capable of developing approximately 80% of their 28-day strength after three days. Meanwhile, the LWACs reinforced with a single fiber showed approximately 90% of their 240-day strength at 28 days, whereas the LWAC containing hybrid fibers demonstrated only 85% of such strength. In other words, the fiber in hybrid form leads to a greater compressive strength after 28 days. Generally, the LWAC gained a higher compressive strength at an early age (0–28 days) in comparison with that at a late age (28–240 days), and the best performance was observed within 0–3 days. This phenomenon might be attributed to the slight internal curing around the LWA in the LWAC mixture. The stored water in the LWA promotes the hydration reaction of the cement, helping at improving the early age curing and consequently increasing the early strength of the LWAC [47]. In addition, the addition of silica fume in the concrete mix can also improve its early compressive strength. During the hydration process, silica fume reacts with calcium hydroxide (CH) crystals to produce a calcium silicate hydrate (C-S-H) gel, which consequently improves the aggregate–cement paste interface [48].

### 3.5. Splitting Tensile and Flexural Strength

**Figure 9:**
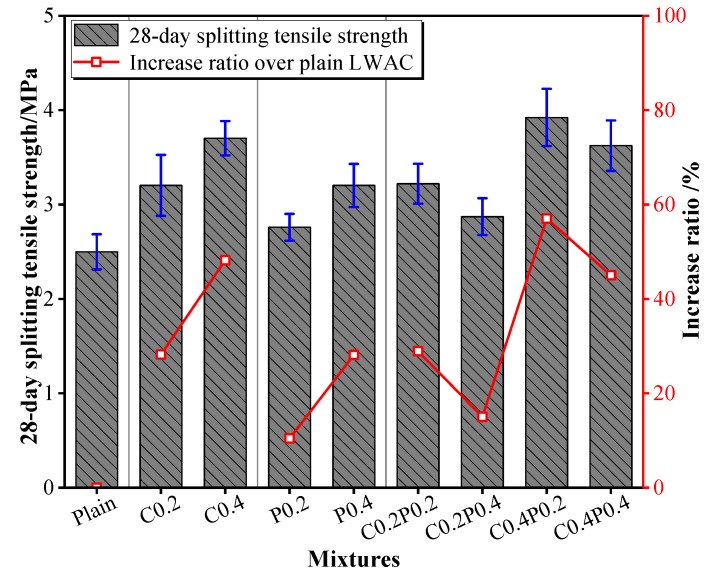
The splitting tensile strength of LWAC.

**Figure 10:**
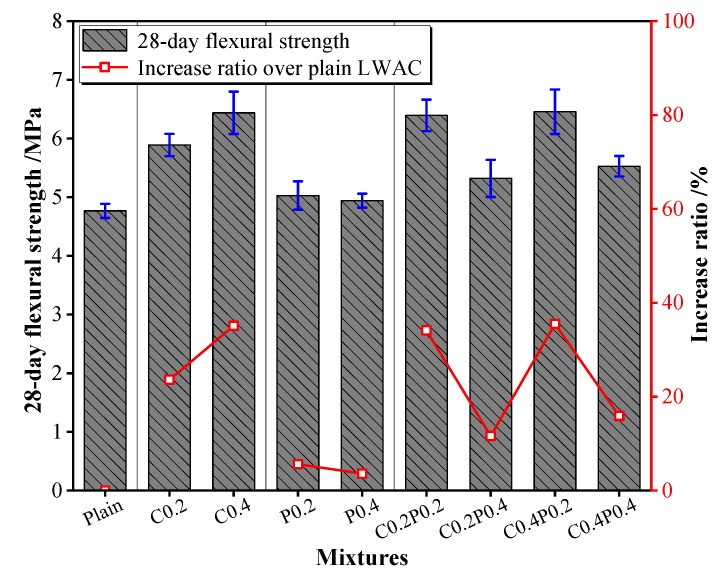
The flexural strength of LWAC.

In this study, splitting tensile and flexural tests were conducted to investigate the tensile behavior of LWAC. The variations in splitting tensile and flexural strengths in response to carbon and/or polypropylene fiber content are shown in Figure 9 and Figure 10. As expected, both the splitting tensile and flexural strengths of the LWAC were continuously improved under any combination of incorporated fibers. The splitting tensile strength increments of single carbon fiber and polypropylene fiber reinforced LWAC ranged from 28.2% to 48.2% and 10.4% to 28.1%. Instead, the enhancement was 15% to 57% for hybrid fiber-reinforced LWAC. As referred to the flexural strength, the improvements of LWAC containing carbon and polypropylene fiber were within the range of 23.6%–35.1% and 3.6%–5.6%, respectively, and the increased percentage of hybrid fiber-reinforced LWAC ranged from 11.6% to 35.5%. Among the hybrid fiber-reinforced concrete, when the carbon fiber content was fixed, both the splitting tensile and flexural strengths decreased with an increase in the amount of polypropylene fiber. It can be concluded that the addition of carbon fiber in the LWAC is more effective compared with that of polypropylene fiber in improving the tensile strength, particularly the flexural strength, owing to the higher elastic modulus of the carbon fiber and better adhesion with the cement paste. Destruction of the carbon fiber can be observed on the fractured surface of the specimens, depicting significant bonding properties of the carbon fiber with cement paste [49]. Furthermore, the highest increment in the splitting tensile and flexural strength was obtained for the C0.4PP0.2 mixture. In this mixture, the fiber hybridization increases the tensile strength of LWAC more than that of the single fiber, showing a positive synergy effect between the carbon and polypropylene fiber. In addition, adding fiber to the concrete results in higher efficiency in improving the flexural strength than the splitting tensile strength. More recently, as Yew et al. [39] stated, a low fiber dosage up to 0.375% was more effective in improving the flexural strength.

**Figure 11:**
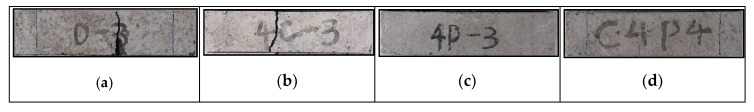
Flexural failure mechanism for LWAC specimens reinforced with: (**a**) no fiber, (**b**) single carbon fiber, (**c**) single polypropylene fiber, (**d**) hybrid fibers.

Figure 11 shows the typical failure mechanism of LWAC specimens, which can directly reflect the fiber reinforcement. For plain and carbon fiber-reinforced LWAC, the fracture surface propagated throughout the entire cross-section accompanied by the breaking of the carbon fiber and a fractured specimen. By contrast, when referring to the polypropylene and hybrid fiber-reinforced LWAC, the fracture surface simply propagated through a portion of the cross-section, which suggests an effective interfacial adhesion between the fibers and cement paste.

## 4. Compressive Stress–Strain Behavior

### 4.1. Stress–Strain Curve

**Figure 12:**
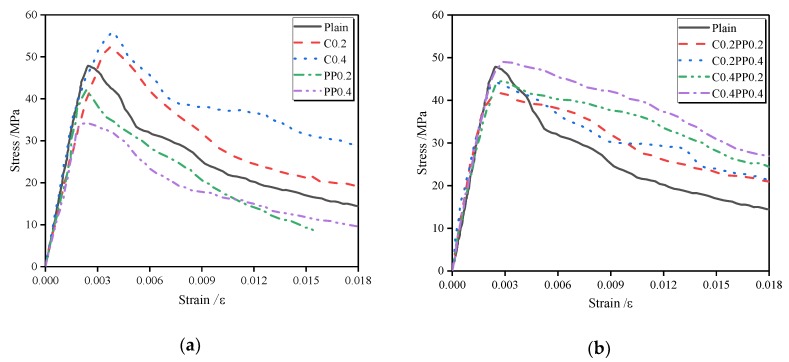
The effect of carbon and/or polypropylene fiber on the compressive stress–strain curves: (**a**) fiber in single form; (**b**) fiber in hybrid form.

Figure 12 shows the compressive stress–strain curve of LWAC reinforced with carbon and polypropylene fibers, which is the average curve provided by the tests performed for each mixture typology. All stress–strain curves can be divided into pre- and post-peak portions corresponding to ascending and descending branches, respectively. The slope of the ascending branch initially changes slightly, and therefore a linear elasticity of the concrete can be assumed. This linearity of the stress–strain curve approached 90% of the ascending branch, which is a larger portion than that of NWC (30%–45%) [50]. During this stage, micro-cracks that formed before the loading will not develop at a relatively low load. As the load increases, the micro-cracks start to propagate stably up to the critical elastic point. The ascending branch gradually deviates from the linearity, and the slope of the curve decreases continuously until it reaches zero at the peak point. The post-peak behavior can be described through a work softening, which is characterized by the descending branch of the curves [51].

The impact of the fibers on the initial behavior of the stress–strain curve was less sensitive in either single or hybrid form, whereas the pattern in the post-peak zone was significantly affected. For LWAC reinforced with a single fiber, the slope of the descending branch is close to that of plain concrete. With the addition of carbon-polypropylene hybrid fibers, the descending branch becomes flat and the slope of this pattern decreases as compared with that of plain concrete. Moreover, it can also be observed that there is a smooth transition from an ascending branch to a descending branch for mixtures with hybrid fibers, indicating that hybridization of the fibers leads to an additional improvement in the energy consumption, and consequently, the brittle behavior of the LWAC can be moderated. The axial compressive strength, the strain at the peak stress, and the elasticity modulus, which are significant parameters for describing the compressive stress–strain curve, are discussed in the following sections.

### 4.2. Characteristics of Stress–Strain Curves

#### 4.2.1. Axial Compressive Strength

**Figure 13:**
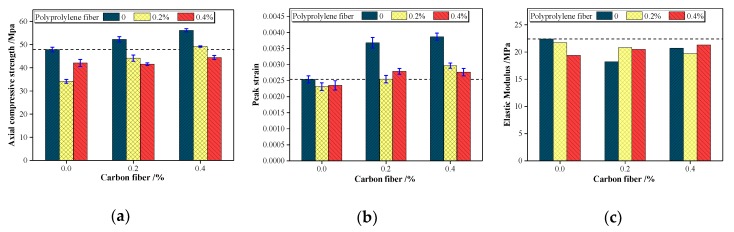
Stress–strain characteristic values for LWAC reinforced with carbon and/or polypropylene fiber: (**a**) axial compressive strength; (**b**) peak strain; (**c**) elastic modulus.

The axial compressive strength is the peak stress obtained from the stress–strain curve. Figure 13a shows the axial compressive strength of LWAC reinforced with carbon and/or polypropylene fibers. The alteration of the axial compressive strength was consistent with that of the compressive strength of the cube, both of which are in line with the change in the fiber combination. The carbon fiber increased the axial compressive strength of the LWAC by 9% and 17%, whereas the opposite effect occurred with the addition of polypropylene fiber (12% and 29% reduction in the axial compressive strength). This enhancement in the compressive strength can be attributed to the load-bearing skeleton of carbon fibers that formed in the cement paste because of the high elastic modulus and tensile strength of carbon fibers. However, the low elastic modulus of polypropylene fibers caused more defects to the compressive strength. Furthermore, applying carbon-polypropylene fiber combinations resulted in a 7%–13% lower axial compressive strength than plain LWAC, except for the case of C0.4PP0.4, which showed a 3% increase. The compressive strength increased with the hybrid fiber content and the adverse effect of polypropylene fiber was modified when combined with carbon fiber.

#### 4.2.2. Peak Strain

The peak strain is taken as the axial strain corresponding to the peak stress on the stress–strain curve, which can be used to quantify the deformability of the mixtures. Figure 13b shows the peak strain of the LWAC reinforced with carbon and/or polypropylene fibers. The peak strain of LWAC was within the range of 0.00231–0.00387, which is larger than the general peak strain of 0.002 for NWC as indicated by the Chinese Standard GB 50010-2010 [52].

The peak strain is considered to increase with an increase in the compressive strength and to be related with the elastic modulus as well as the fiber combination. Single carbon fiber-reinforced LWAC showed a peak strain of 0.00368 and 0.00387, which are much higher values than 0.00234 for plain LWAC. However, a slight decrease in peak strain was observed with the addition of a single polypropylene fiber. The main reason for the change in the peak strain can be attributed to the influence of the elastic modulus, and the peak stress also played a role. Both an increase in the peak stress and a decrease in the elastic modulus lead to a large deformation, and further produced an enhanced peak strain in the concrete. Similarly, Xiao et al. [53] reported that a 20% increase in the peak strain is due to the 40% reduction in the elastic modulus, as compared with that of the control.

#### 4.2.3. Elastic Modulus

Following the China standard GB/T 50081-2019 [31], the elastic modulus is defined as the secant modulus obtained from the experimental stress–strain curve at the point of 1/3 peak stress. It has been suggested that the structural deformability and stiffness of the concrete member are directly affected by the elastic modulus [54]. As shown in Figure 13c, the elastic modulus of plain and fiber-reinforced LWAC was within the range of 18.2–22.4 GPa, which is much lower than that of NWC. As suggested by CEB-FIP Model Code 2010, the tangent elastic modulus of NWC is varied from 36.3 to 40.7 GPa in accordance with the compressive strength of 40–60 MPa [55]. The elastic modulus of concrete depends substantially on the composition of concrete, and as a result porous LWA, resulting in an increase in the deformability characteristic of concrete, is directly responsible for the lower elastic modulus. Moreover, a slight reduction in elastic modulus can be observed with the addition of fibers. However, it is reported that the elastic modulus should not be influenced by the fiber itself because the discrete fiber cannot provide an obvious compression resistance [56]. The decrease in elastic modulus is attributed to the interference of the fibers on the compactness of the concrete. Previously, a similar reduction in elastic modulus was claimed by Suksawang [56] and Neves and Almeida [57].

### 4.3. Ductility

**Figure 14:**
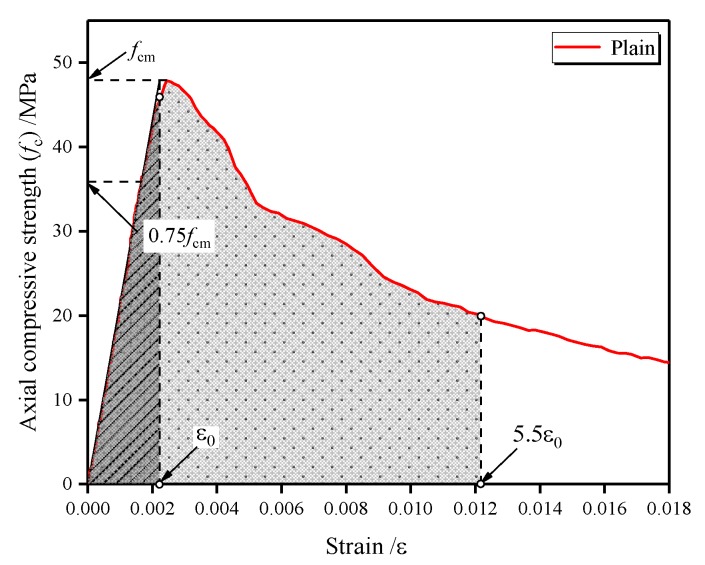
Schematic representation of ductility.

The post-peak ductility of concrete, related to the deformability, can be quantitatively evaluated based on the ductility index. In accordance with ASTM C1018-92 [58], the ductility index (*I*_10_) can be defined as the ratio of the integral of the stress–strain curve at up to 5.5-times the specified strain of the mixture to the integral of the curve up to the specified strain of the mixture (Equation (3)). The specified strain, *ε*_0_, is the strain corresponding to the elastic behavior limit, which can be determined based on the 3/4 rule. In the stress–strain curve, the ascending branch is secant at 75% of the peak stress and extrapolated until reaching the peak stress to determine the specified strain, as plotted in Figure 14 [59]. This definition provides a value of 10 for *I*_10_ corresponding to a perfect elasto-plastic material and a value of 1 corresponding to a perfect elastic-brittle material [60].

(3) $$I_{10}=\frac{\int_{0}^{5.5\varepsilon_{0}}f_{c}\left(\varepsilon \right)d\varepsilon }{\int_{0}^{\varepsilon_{0}}f_{c}\left(\varepsilon \right)d\varepsilon }$$

**Figure 15:**
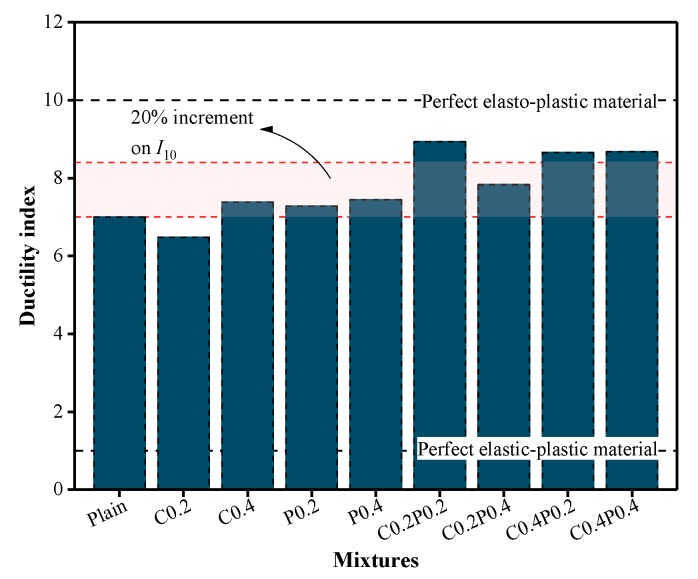
Ductility index for plain and fiber-reinforced LWAC.

As shown in Figure 15, the ductility index of the plain LWAC was 7.01, whereas, for LWAC reinforced with single and hybrid fibers, the ductility index was within the range of 6.48–7.44 and 7.83–8.94, respectively. For LWAC subjected to compression, the crack penetrates through the LWA instead of avoiding the LWA, thus the deformable characteristic of LWA can be fully used, leading to an increment in the ductility of LWAC. Regarding the change in ductility index, the contribution of a single fiber was highly limited in improving the deformability after the peak stress when compared with the plain LWAC. The addition of hybrid fibers resulted in a much higher ductility index by 12%–27% than that of plain LWAC, and the largest increase was obtained by a C0.2PP0.2 mixture. This result indicates an improved post-peak ductility behavior for LWAC reinforced with hybrid fibers, demonstrating a significant positive synergy between the carbon and polypropylene fiber.

### 4.4. Toughness

The influence of fibers on the energy-absorbing capacity can be determined by the toughness, which is calculated based on the integral of the stress–strain curve up to the specified strain. However, because the toughness is affected by the incorporation of fiber as well as the compressive strength, the toughness ratio (ratio of toughness-to-specified strain and compressive strength *f*_cm_) may be considered a more efficient way to evaluate the energy-absorbing capacity, as proposed by Nataraja et al. [61] and Jang and Yun [62]. Meanwhile, the toughness ratio is recognized as a good measure for the relative absorbed energy. The specified strain values were three- and five-times the ultimate strain of 0.003 recommended by the ACI standard [63]. It was reported by Fenella and Naaman [64] that the specified strain of 0.015 is sufficient to reflect the characteristic of the post-peak stress–strain behavior.

(4) $$TR_{i}=\frac{\int_{0}^{\varepsilon_{i}}f_{c}\left(\varepsilon \right)d\varepsilon }{f_{cm}\varepsilon_{i}}=\frac{TF_{i}}{f_{cm}\varepsilon_{i}}$$

**Figure 16:**
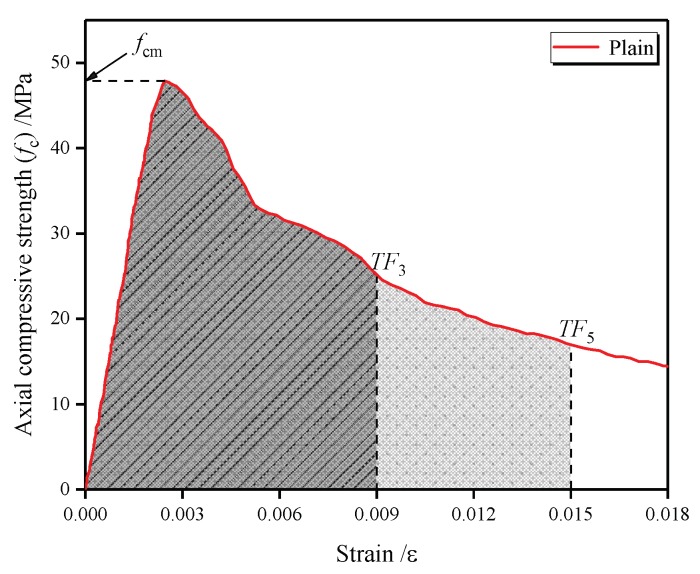
Schematic representation of toughness.

Herein, *TR*_3_ and *TR*_5_ are the compressive toughness ratios up to a strain of *ε*_3_ and *ε*_5_, respectively; *ε*_3_ and *ε*_5_ are the strain corresponding to specified strain of 0.009 and 0.015, respectively; and *f_cm_* is the axial compressive strength. As illustrated in Figure 16, *TR*_3_ and *TR*_5_ are the integral of stress–strain curve before the specified strain of 0.009 and 0.015, respectively.

**Figure 17:**
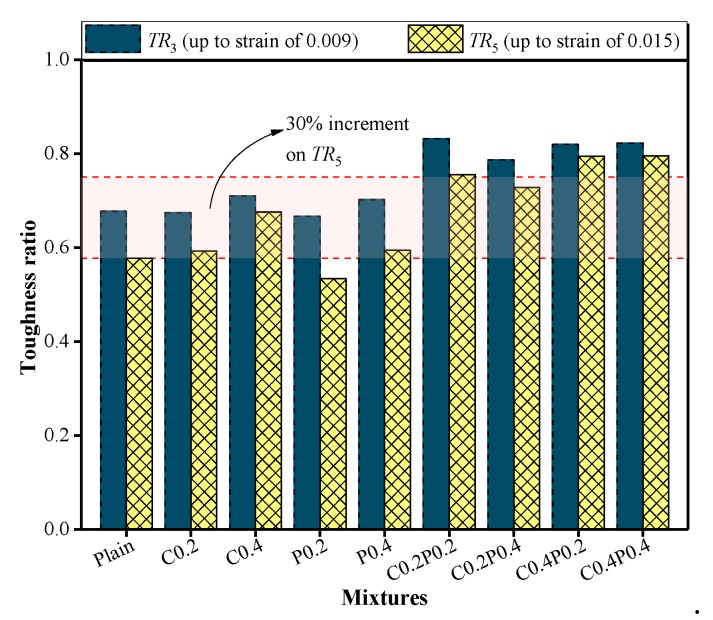
Toughness ratio of plain and fiber-reinforced LWAC.

The results of the toughness ratio obtained at different combinations of carbon and/or polypropylene fibers are shown in Figure 17. As indicated, the toughness ratio results, *TR*_3_ and *TR*_5_, calculated using Equation (3), have a similar tendency. It seems reasonable to suggest that this equation can be used to evaluate the complete post-peak behavior. The toughness ratio of LWAC reinforced with a single fiber was observed to be close to that of plain LWAC, except for the C0.4 mixture. The C0.4 mixture shows *TR*_3_ and *TR*_5_ of 0.724 and 0.690, respectively, which is 7% and 19% higher than in the plain mixture. The addition of a single carbon fiber can improve the toughness of LWAC as the fiber reaches up to 0.4%. This is due to the restricted bridging effect of the fiber because the fiber skeleton in the concrete matrix is not formed when the fiber content is low.

After adding the hybrid carbon-polypropylene fibers, *TR*_3_ and *TR*_5_ of the LWAC increased by 16%–23% and 26%–37%, respectively. By comparing mixtures C0.2PP0.2 with C0.2PP0.4 and C0.4PP0.2 with C0.4PP0.4, a decrease in the toughness ratio can be observed under an increase in the polypropylene fiber content. Therefore, the increment of the polypropylene fiber reduces the effect of the hybrid fibers. However, such a reduction is significantly lower compared with an increase in the addition of the hybrid fibers. Taking the *TR*_5_ value as an example, C0.2PP0.4 and C0.4PP0.4 generated an additional 26% and 38% toughness ratio when compared with the plain mixture. Therefore, the LWAC reinforced with hybrid fibers showed a much greater toughness ratio than those with a single fiber, which indicates their higher energy-absorbing capacity. Moreover, the increase in toughness ratio can be explained by the flat descending part of the stress–strain curve. The combination of carbon and polypropylene fiber was highly effective on the post-peak behavior, resulting in a more ductile behavior and a reduction in the brittleness of the LWAC during the compression.

## 5. Conclusions

In this study, both the single and hybrid effects of carbon and polypropylene fibers with different volume fractions on LWAC were investigated, and the following was determined:

(1) All nine LWAC mixtures exhibited acceptable workability and segregation resistance. The addition of carbon and/or polypropylene fibers to the LWAC decreased the slump value but improved the segregation resistance. The stability of LWA in the concrete mixture was significantly affected by the composition and the workability of the concrete mixture.

(2) Both the splitting tensile and flexural strengths were significantly improved when the LWAC was reinforced with carbon and/or polypropylene fibers, particularly in a hybrid form corresponding to increments of 15%–57% and 11.6%–35.5%, respectively. By contrast, the presence of a single polypropylene fiber and hybrid fibers resulted in a marginal decrease in the specific and compressive strengths.

(3) LWAC reinforced with both single and hybrid fibers displayed a similar linearity branch of the stress–strain curve. However, the pattern in the post-peak zone of the LWAC exhibited a significant difference among the single and hybrid fibers. Compared with single fiber-reinforced LWAC, the hybrid fiber form led to a superior modification in the post-peak behavior of the curve.

(4) Hybrid fiber-reinforced LWAC generated an additional 26%–37% toughness ratio as well as a 12%–27% ductility index compared with plain LWAC, presenting a pronounced effect on the energy-absorbing capacity and post-peak behavior. The hydration of carbon and polypropylene fibers can effectively improve the toughness and ductility of LWAC, consequently moderating its brittle behavior.

(5) Compared with single fiber-reinforced LWAC, the fiber in hybrid form led to an additional modification of the tensile strength, toughness, and post-peak ductility, presenting a positive synergy effect of the hybrid carbon and polypropylene fibers.

## Figures and Tables

**Table 1:** Fiber addition of lightweight aggregate concrete (LWAC) mixtures.

Fiber/(%)	–	Carbon Fiber		Polypropylene Fiber		Basalt/Polypropylene Hybrid Fibers			
	–	0.2	0.4	0.2	0.4	0.2/0.2	0.2/0.4	0.4/0.2	0.4/0.4
Mix code	Plain	C0.2	C0.4	PP0.2	PP0.4	C0.2PP0.2	C0.2PP0.4	C0.4PP0.2	C0.4PP0.4

**Table 2:** Mix composition of plain LWAC/(kg/m^3^).

Cement	Silica Fume	Fly Ash	LWA ^1^	Sand	Water	Superplasticizer	w/b ^2^
440	44	66	578	667	165	5.2	0.3

^1^ Lightweight aggregate; ^2^ water-to-binder ratio.

**Table 3:** Properties and grading of LWA.

Apparent Density/(kg/m^3^)	Bulk Density/(kg/m^3^)	Total Porosity/(%)	1 h/24 h Water Absorption/(%)	Crushing Strength/MPa	Aggregate Volume Fraction/(%)		
					2.36–5 mm	5–10 mm	10–16 mm
1512	860	43.12	2.2/2.6	6.9	11	68	21

**Table 4:** Properties of investigated fibers.

Fiber Species	Length/mm	Diameter/μm	Shape	Density/(kg/m^3^)	Fracture Elongation/(%)	Elasticity Modulus/GPa	Tensile Strength/MPa
Carbon (C)	8–10	7	Straight round bundled filaments	1800	2.1	240	>4000
Polypropylene (PP)	15–22	80	Straight round bundled filaments	910	17	4.2	>400

**Table 5:** Chemical composition of binder.

Binder	CaO/(%)	SiO_2_/(%)	Al_2_O_3_/(%)	Fe_2_O_3_/(%)	MgO/(%)	SO_3_/(%)	K_2_O/(%)	Na_2_O/(%)	LOI ^2^/(%)	SG ^3^/(g/cm³)
Cement	62.81	20.36	5.67	3.84	2.68	2.51	0.87	0.19	1.07	3.15
SF ^1^	0.48	92.83	0.68	1.17	1.24	0.40	1.21	0.95	1.04	2.7
Fly ash	7.74	48.40	27.13	8.04	2.47	1.48	1.61	2.35	0.78	2.6

^1^ SF, silica fume; ^2^ LOI, loss on ignition; ^3^ SG, specific gravity.

**Table 6:** Test item and specimen dimensions for each concrete mixture.

Test	Specimen Size/mm^3^	Number of Specimens	Test Age
Cube compressive strength	100 × 100 × 100	3 × 6	3, 7, 28, 60, 120, 240
Splitting tensile strength	100 × 100 × 100	3	28
Flexural tensile strength	100 × 100 × 400	3	28
Stress–strain behavior	100 × 100 × 300	2	28

**Table 7:** Physical properties of LWAC with single and hybrid fibers.

Mix Code	Slump/mm	Slump Type	Demolded Density/(kg/m^3^)	Oven-Dry Density/(kg/m^3^)
Plain	255	Collapse	2049	1849
C0.2	214	Collapse	1995	1825
C0.4	102	True	2042	1844
PP0.2	250	Collapse	1886	1730
PP0.4	235	Collapse	1871	1728
C0.2PP0.2	212	True	1892	1724
C0.2PP0.4	160	True	1932	1762
C0.4PP0.2	65	True	1927	1751
C0.4PP0.4	75	True	1993	1808

**Table 8:** Distribution of coarse LWA for LWAC and corresponding segregation parameters.

Mixture ID	Layer	LWA/g	*SC*/(%)	*MI*/(%)
Plain	Top	1116	1.381	2.762
	Bottom	1056		
C0.2	Top	1093	1.075	2.150
	Bottom	1047		
C0.4	Top	1028	0.442	0.883
	Bottom	1010		
PP0.2	Top	1087	1.346	2.692
	Bottom	1030		
PP0.4	Top	1180	0.840	1.680
	Bottom	1141		
C0.2PP0.2	Top	1040	1.206	2.413
	Bottom	991		
C0.2PP0.4	Top	1020	0.746	1.493
	Bottom	990		
C0.4PP0.2	Top	1010	0.374	0.748
	Bottom	995		
C0.4PP0.24	Top	1017	0.347	0.693
	Bottom	1003		

**Table 9:** Mechanical property test results of LWAC.

Mixture Code	Compressive Strength/(MPa)						Splitting Tensile Strength/(MPa)	Flexural Strength/(MPa)
	3-Day	7-Day	28-Day	60-Day	120-Day	240-Day		
Plain	49.28 (0.036)	50.23 (0.041)	58.24 (0.030)	59.54 (0.057)	61.44 (0.052)	62.90 (0.041)	2.50 (0.075)	4.77 (0.025)
C0.2	44.32 (0.061)	47.21 (0.031)	57.99 (0.033)	59.84 (0.036)	60.82 (0.032)	61.45 (0.037)	3.20 (0.101)	5.89 (0.032)
C0.4	48.21 (0.024)	52.06 (0.025)	67.11 (0.031)	68.39 (0.035)	72.10 (0.042)	73.59 (0.052)	3.70 (0.095)	6.44 (0.056)
PP0.2	39.33 (0.069)	41.06 (0.055)	49.64 (0.047)	50.69 (0.067)	55.63 (0.008)	56.02 (0.038)	2.76 (0.051)	5.03 (0.048)
PP0.4	32.33 (0.033)	33.91 (0.053)	40.99 (0.064)	42.70 (0.027)	44.25 (0.072)	45.35 (0.058)	3.20 (0.071)	4.94 (0.092)
C0.2PP0.2	38.16 (0.024)	42.17 (0.061)	48.26 (0.019)	51.58 (0.012)	54.66 (0.037)	57.08 (0.030)	3.22 (0.094)	6.39 (0.042)
C0.2PP0.4	40.10 (0.036)	47.30 (0.039)	50.65 (0.030)	54.89 (0.020)	56.86 (0.057)	58.96 (0.044)	2.87 (0.068)	5.32 (0.096)
C0.4PP0.2	39.28 (0.046)	43.68 (0.050)	51.36 (0.047)	54.78 (0.023)	56.07 (0.013)	58.67 (0.025)	3.92 (0.101)	6.46 (0.059)
C0.4PP0.4	44.78 (0.096)	51.82 (0.017)	56.88 (0.028)	60.13 (0.016)	63.62 (0.043)	66.72 (0.059)	3.62 (0.074)	5.53 (0.032)
